# 非小细胞肺癌原发灶和转移灶EGFR和KRAS状态比较的*meta*分析

**DOI:** 10.3779/j.issn.1009-3419.2010.09.09

**Published:** 2010-09-20

**Authors:** 琤波 韩, 华伟 邹, 洁韬 马, 洋 周, 健竹 赵

**Affiliations:** 110022 沈阳，中国医科大学附属盛京医院第一肿瘤科 Department of Oncology, Shengjing Hospital of China Medical University, Shenyang 110022, China

**Keywords:** 肺肿瘤, 原发灶, 转移灶, 表皮生长因子受体, KRAS, 突变, Lung neoplasms, Primary tumor, Metastases, Epidermal growth factor receptor, KRAS, Mutation

## Abstract

**背景与目的:**

非小细胞肺癌*EGFR*和*KRAS*基因状态对肺癌一线靶向治疗的选择尤为关键，而原发肿瘤和转移瘤之间可能存在不同的*EGFR*和*KRAS*基因状态。本研究旨在系统评价比较配对的原发肺癌灶和转移灶*EGFR*和*KRAS*基因状态以指导临床实践。

**方法:**

通过Pubmed数据库检索所有符合检索条件的文献，末次检索日期2010年5月10日，根据纳入和排除标准进一步筛选。采用*meta*分析方法比对肺癌原发灶和转移灶中*EGFR*基因突变、扩增、EGFR蛋白表达和*KRAS*基因突变状态之间的差异。

**结果:**

14篇文献纳入*meta*分析，具有配对的原发灶和转移灶，598例 *vs* 598例。原发灶中EGFR蛋白表达和*KRAS*基因的突变频率高于转移灶，RR分别为1.13(95%CI: 0.98-1.31, *P*=0.09)和1.39(95%CI: 0.95-2.03, *P*=0.09)。转移灶中*EGFR*的基因拷贝数高于原发灶，RR=0.74(95%CI: 0.53-1.02, *P*=0.06)。*EGFR*基因在原发灶和转移灶中的突变频率无统计学差异(*P*=0.31)。原发灶和转移灶基因状态不一致率分别为：*EGFR*突变率为17.09%；*EGFR*扩增率为27.07%；EGFR蛋白表达率为27.84%；*KRAS*突变率为25.91%。

**结论:**

肺癌原发灶和相应转移灶中*EGFR*基因突变较*KRAS*基因状态更为稳定，原发灶中*KRAS*基因突变更能反映肺癌周身癌灶*KRAS*基因特征，单独对转移灶做KRAS状态分析可能会引入更多抵抗EGFR酪氨酸激酶抑制剂治疗的患者。联合检测原发灶中*EGFR*和*KRAS*基因突变可作为肺癌靶向治疗的疗效预测指标。

## Abstract (en)

**Background and objective:**

Epidermal growth factor receptor (EGFR) and KRAS status were particularly critical for the choice of first-line targeted therapy of non-small cell lung cancer (NSCLC), while the primary tumor and metastases might be different in the *EGFR* and *KRAS* gene status. The aim of this pooled analysis is to compare EGFR and KRAS status in matching primary NSCLC and metastases and further to guide clinical practice.

**Methods:**

Systematic computerized searches of the Pubmed and Medline databases (up to May 10, 2010) meeting specified search criteria were performed, followed by a further screening according to inclusive and exclusive criteria.

**Results:**

Fourteen articles were selected into the final *meta*-analysis with paired primary and metastatic cases of 598. Expression level of EGFR protein and mutation frequency of *KRAS* gene in primary tumors were higher than that in metastases, relative risk (RR)=1.13 (95%CI: 0.98-1.31, *P*=0.09) and RR=1.39 (95%CI: 0.95-2.03, *P*=0.09), respectively. *EGFR* gene copy number in metastases was higher than that in primary tumor, RR=0.74 (95%CI: 0.53-1.02, *P*=0.06). There was no statistically significant difference of *EGFR* mutation frequency in primary tumors and metastases (*P*=0.31). The discordant rate in primary and metastases was 17.09% for *EGFR* mutation, 27.07% for *EGFR* amplification, 27.84% for EGFR protein expression and 25.91% for *KRAS* mutation.

**Conclusion:**

The systematic analysis showed that the *EGFR* mutation status in primary lung cancer and corresponding metastases was more stable than *KRAS* gene. *KRAS* mutation in primary lung cancerous foci seems to better reflect systemically cancerous genetic characteristics of *KRAS* gene. Determination of *KRAS* gene status based merely on metastatic foci might lead to more resistant selections of EGFR tyrosine kinase inhibitor (TKI) therapy. Combined detection of *EGFR* and *KRAS* mutation from primary NSCLC foci might serve as a better predictive biomarker for anti-EGFR targeted therapy.

肺癌是全世界最常见的恶性肿瘤，也是癌症相关死亡首要死因。大约70%的非小细胞肺癌(non-small cell lung cancer, NSCLC)患者在初始诊断时即为局部晚期或转移性疾病^[1]^。NSCLC具有多基因改变积聚的特点^[2, 3]^，较为常见的基因突变包括*p53*、表皮生长因子受体(epidermal growth factor receptor, *EGFR*)和*KRAS*等^[4]^。EGFR酪氨酸激酶抑制剂(tyrosine kinase inhibitor, TKI)，如吉非替尼和厄洛替尼，作用于EGFR胞内段酪氨酸激酶区ATP结合域。当癌细胞发生EGFR外显子18、19和21突变激活时，可显著提高局部晚期或转移性NSCLC患者的客观缓解率，延长总生存期^[5]^。国外研究^[6-8]^显示，*KRAS*突变见于多达30%的肺腺癌，是NSCLC的预后不良因素以及EGFR-TKI疗效不佳的预测指标。若干研究^[9-11]^显示，*EGFR*基因扩增、mRNA和蛋白表达水平也可用来预测TKI治疗反应。60%-80%的NSCLC存在EGFR表达，而其一度也被认为是EGFR单克隆抗体(如西妥昔单抗)的疗效预测指标。西妥昔单抗作用于EGFR胞外域，临床试验显示西妥昔单抗联合标准化疗显著改善晚期NSCLC患者的总生存^[12]^。值得关注的是，不表达EGFR和部分EGFR野生型的NSCLC患者也可能从EGFR-TKI或EGFR单克隆抗体治疗中获益^[13]^。有研究者^[14]^提出可能是由于*EGFR*突变或表达状态的确定往往是通过原发灶手术切除或活检获得组织进行分析，而大量的分子改变可能发生在转移过程中。即原发肿瘤和转移瘤之间可能存在不同的*EGFR*和*KRAS*基因状态。因此，我们通过对相关文献进行*meta*分析系统评价比较配对的原发肺癌灶和转移灶*EGFR*和*KRAS*基因状态以指导临床实践。

## 1 材料和方法

### 1.1 文献资料来源

通过Pubmed数据库检索所有符合以下检索词的文献。串联检索关键词：primary NSCLC和metastases，加入并列检索词：EGFR或KRAS。中文检索通过中国期刊全文数据库，检索关键词：肺癌、EGFR或KRAS。末次检索日期为2010年5月10日。

### 1.2 文献资料的纳入标准

未曾接受过EGFR-TKI治疗且具有转移淋巴结或其它肺外转移灶的NSCLC患者。研究对象必须包含配对的原发灶和转移灶，原发灶和转移灶组织获取方法不限，可以为支气管镜、穿刺或手术后组织。检测指标具备以下任何之一者纳入本研究：①*EGFR*突变检测位点至少包括外显子19和21；②*KRAS*突变位点至少包括密码子12和13；③EGFR蛋白表达采用统一可评价的免疫组织化学(IHC)方法和标准；④*EGFR*基因拷贝数(gene copy number)采用统一的荧光原位杂交(FISH)方法和判断标准。

### 1.3 文献资料的排除标准

具有以下之一者排除该项研究或该病例研究：①曾接受过EGFR-TKI治疗的NSCLC；②无配对原发灶和转移灶，或原发灶配对外周血标本；③无明确病理诊断；④肺转移癌。具有以下之一者排除该指标研究：①文献资料中未明确*EGFR*或*KRAS*的突变特征，或不符合观察的最少检测位点；②EGFR蛋白表达、基因扩增无统一的判定标准。

### 1.4 *meta*分析指标及相关定义

*meta*分析比对NSCLC原发灶和转移灶EGFR、KRAS状态，包括：①*EGFR*基因突变；②*EGFR*基因拷贝数；③EGFR蛋白表达；④*KRAS*基因突变。

所有纳入的研究采用统一的可评价方法。①EGFR FISH阳性定义：具有高水平的多倍体(40%以上细胞中≥4个拷贝数)或是基因扩增。基因扩增定义为出现紧密基因簇(tight gene clusters)，每个细胞中基因/染色体之比≥2，或是在≥10%分析细胞中每个细胞≥15个基因拷贝^[15]^。②EGFR蛋白IHC阳性定义：10%以上肿瘤细胞膜染色定义为阳性^[16]^。③*EGFR*和*KRAS*突变采用直接测序法。

### 1.5 统计分析

采用χ^2^检验进行各研究间的异质性检验，假设齐性检验的α值为0.1。若异质性检验提示各研究结果间异质性不显著(*P*＞0.10)，则采用*Mantel Haenszel*固定效应模型计算合并的相对危险度(relative rate, RR)值及其95%可信区间(confidence interval, CI)；反之，若同质性检验结果提示各研究异质性显著(*P*≤0.10)，则采用*Dersimonian*-*Laird*随机效应模型(*D*-*L*法)计算合并后的RR值及其95%CI。应用*meta*分析软件包RevMan 4.2实现上述计算，以纳入*meta*分析的各研究的1/SE代替样本量作为纵坐标，以各研究的OR值作为横坐标绘制漏斗图，直观上评估发表偏倚的影响。另外以失安全系数(fail-safe number, N_fs_)来估计发表偏倚对在*P*=0.05水平有统计学意义的合并检验结果的影响。N_fs0.05_=(ΣZ/1.64)^2^-*K*，此值越大说明发表偏倚的影响越小，结论的可靠性越好。

## 2 结果

### 2.1 文献基本情况

中国期刊全文数据库未检出相关文献，通过Pubmed共检索到31篇相关文献，经过筛选纳入*meta*分析共14篇，具有配对的原发灶和转移灶病例共598例 *vs* 598例。14篇所对比的*EGFR*基因突变、扩增、EGFR蛋白表达和*KRAS*基因突变均归一为统一的方法评价。其中*EGFR*突变相关7篇，配对357例；*KRAS*突变相关6篇，配对193例；EGFR蛋白表达相关6篇，配对195例；EGFR扩增相关4篇，配对133例。纳入研究的具体数据参见表 1-表 4。

**1:** 纳入的NSCLC *EGFR*突变研究 Eligible studies for *EGFR* mutation of NSCLC

*EGFR* mutation	Pairs	Primary NSCLC			Metastases			Inconsistency	
		*n*	Percent		*n*	Percent		*n*	Percent
Matsumoto S^[17]^	19	6	31.58%		12	63.16%		11	57.89%
Cortot AB^[18]^	21	0	0.00%		0	0.00%		0	0%
Kalikaki A^[19]^	25	5	20.00%		3	12.00%		6	24.00%
Daniele L^[15]^	28	0	0.00%		0	0.00%		0	0%
Gow CH^[20]^	67	18	26.87%		26	38.81%		26	38.81%
Park S^[21]^	101	30	29.70%		28	27.72%		12	11.88%
Schmid K^[22]^	96	4	4.17%		4	4.17%		6	6.25%
Sum	357	63	17.17%		73	19.89%		61	17.09%

**2:** 纳入的NSCLC EGFR扩增研究 Eligible studies for EGFR amplification of NSCLC

EGFR amplification	Pairs	Primary NSCLC			Metastases			Inconsistency	
		*n*	Percent		*n*	Percent		*n*	Percent
Italiano A^[23]^	30	16	53.33%		12	40.00%		7	23.33%
Bozzetti C^[24]^	28	10	35.71%		17	60.71%		9	32.14%
Daniele L^[15]^	35	10	28.57%		16	45.71%		8	22.86%
Monaco SE^[25]^	40	3	7.50%		8	20.00%		11	27.50%
Sum	133	39	28.67%		53	38.97%		35	27.07%

**3:** 纳入的NSCLC EGFR蛋白表达研究 Eligible studies for EGFR protein expression of NSCLC

EGFR protein expression	Pairs	Primary NSCLC			Metastases			Inconsistency	
		*n*	Percent		*n*	Percent		*n*	Percent
Italiano A^[23]^	30	20	66.67%		16	53.33%		10	33.33%
Badalian G^[26]^	11	4	36.36%		7	63.64%		6	54.55%
Kalikaki A^[19]^	19	11	57.89%		11	57.89%		8	42.11%
Gomez-Roca^[16]^	49	38	77.55%		28	57.14%		16	32.65%
Rao C^[27]^	47	36	76.60%		37	78.72%		5	10.64%
Watzka SB^[28]^	39	27	69.23%		21	53.85%		12	30.77%
Sum	195	125	71.02%		109	61.93%		57	29.23%

**4:** 纳入的非小细胞肺癌*KRAS*突变研究 Eligible studies for *KRAS* mutation of NSCLC

*KRAS* mutation	Pairs	Primary NSCLC			Metastases			Inconsistency	
		*n*	Percent		*n*	Percent		*n*	Percent
Badalian G^[28]^	11	3	27.27%		3	27.27%		4	36.36%
Cortot AB^[18]^	21	3	14.29%		4	19.05%		6	28.57%
Kalikaki A^[19]^	25	5	20.00%		5	20.00%		6	24.00%
Schmid K^[22]^	96	28	29.17%		20	20.83%		25	26.04%
Monaco SE^[25]^	40	11	27.50%		4	10.00%		9	22.50%
Sum	193	50	25.91%		36	18.65%		50	25.91%

### 2.2 NSCLC原发灶和转移灶*EGFR*和*KRAS*基因状态

同质性检验显示各指标研究组间*P*＞0.10，选择固定效应模型计算合并的RR值及其95%CI。原发灶中EGFR蛋白表达高于转移灶，71.02% *vs* 61.93%，RR=1.13(95%CI: 0.98-1.31, *P*=0.09)；转移灶中*EGFR*的基因拷贝数高于原发灶，38.97% *vs* 28.67%，RR=0.74(95%CI: 0.53-1.02, *P*=0.06)。而*KRAS*基因的突变频率在原发灶中高于其转移灶，25.91% *vs* 18.65%，RR=1.39(95%CI: 0.95-2.03, *P*=0.09)。*EGFR*基因在原发灶和转移灶中的突变频率无统计学差异，17.17% *vs* 19.89%，RR=0.86(95%CI: 0.65-1.15, *P*=0.31)，参见图 1-图 4。漏斗图直观显示*EGFR*和*KRAS*基因突变呈倒漏斗图形，且对称性良好；而*EGFR*基因扩增和蛋白表达研究对称性较差，N_fs_分别为9.44、10.48。漏斗图参见图 5。

**1:**
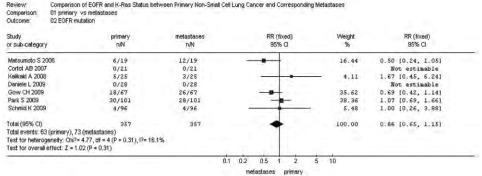
原发灶和转移灶*EGFR*基因突变*meta*分析森林图 Forest graph of *EGFR* mutation *meta*-analysis between primary NSCLC and metastases

**2:**
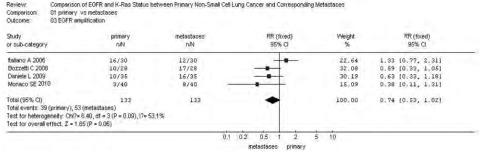
原发灶和转移灶*EGFR*基因扩增*meta*分析森林图 Forest graph of *EGFR* amplification *meta*-analysis between primary NSCLC and metastases

**3:**
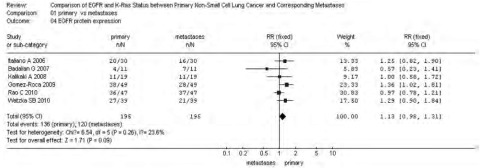
NSCLC原发灶和转移灶EGFR蛋白表达*meta*分析森林图 Forest graph of EGFR protein expression *meta*-analysis between primary NSCLC and metastases

**4:**
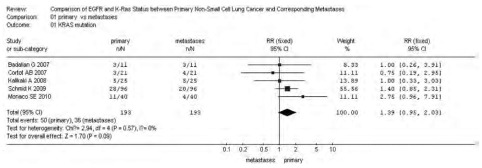
NSCLC原发灶和转移灶*KRAS*基因突变*meta*分析森林图 Forest graph of *KRAS* mutation *meta*-analysis between primary NSCLC and metastases

**5:**
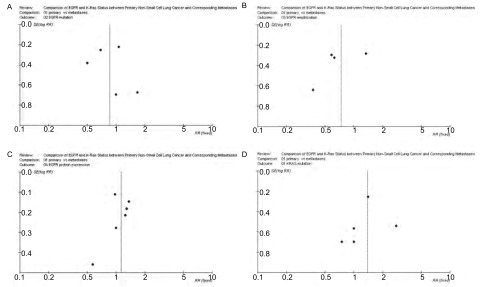
纳入的NSCLC原发灶和转移灶EGFR或KRAS状态研究漏斗图。A：*EGFR*突变；B：EGFR扩增；C：EGFR蛋白表达；D：*KRAS*突变。 Funnel plot of eligible studies for EGFR or KRAS status in NSCLC. A: *EGFR* mutation; B: EGFR amplification; C: EGFR protein expression; D: *KRAS* mutation.

### 2.3 NSCLC原发灶和转移灶*EGFR*和*KRAS*基因符合情况

*EGFR*和*KRAS*基因状态在原发灶和转移灶中的不一致率分别为：*EGFR*基因突变，17.09%(0-57.89%)；EGFR扩增，27.07%(22.86%-32.14%)；EGFR蛋白表达，27.84%(10.64%-54.55%)；*KRAS*基因突变，25.91% (22.50%-36.36%)。森林图(图 6)显示，*EGFR*和*KRAS*基因状态或蛋白表达情况在原发灶和转移灶中一致的较不一致的明显占优(*P*＜0.01)。

**6:**
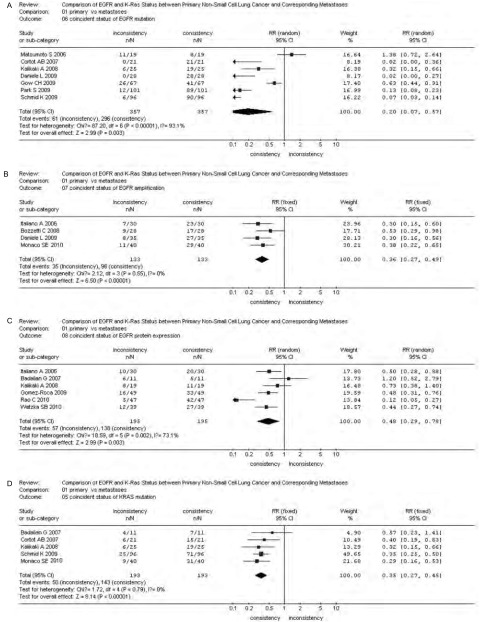
非小细胞肺癌原发灶和转移灶EGFR或KRAS状态符合情况*meta*分析森林图。A：*EGFR*突变；B：EGFR扩增；C：EGFR蛋白表达；D：*KRAS*突变。 Forest graph of coincident status *meta*-analysis of EGFR or KRAS between primary NSCLC and metastases. A: *EGFR* mutation; B: EGFR amplification; C: EGFR protein expression; D: *KRAS* mutation.

## 3 讨论

*EGFR*激活突变与75%-95%的EGFR-TKI治疗的客观有效率相关，而*KRAS*突变与EGFR-TKI敏感性缺乏相关^[6-8]^。也有研究^[9-11]^发现*EGFR*基因扩增和蛋白表达可以作为吉非替尼临床获益的预测标志。研究^[23]^显示，肺癌在分子水平上即便在同一肿瘤中也常常表现出异质性，且在肿瘤进展和转移过程中，*EGFR*突变、表达以及基因拷贝数往往也是不稳定的，即转移灶与原发肿瘤的基因状态往往不一致。而我们通过7项配对研究的*meta*分析显示，NSCLC原发灶和转移灶*EGFR*突变率基本一致，RR=0.86 (*P*=0.31)，突变状态符合率达82.9%，森林图显示*EGFR*基因状态一致的NSCLC明显占优(*P*=0.003)。结果提示*EGFR*突变状态在原发灶和相应转移灶中应是比较保守的。

通过对*KRAS*基因突变的*meta*分析发现，原发灶*KRAS*基因的突变率明显高于转移灶(OR=1.39, 95%CI：0.95-2.03, *P*=0.09)。原发灶和转移灶*KRAS*基因状态一致率为74.9%，*KRAS*基因状态一致的NSCLC仍然占优势。尽管*KRAS*突变似乎与NSCLC早期发生相关，但不能排除*KRAS*突变在肿瘤进展过程中消失^[29]^。这或许可以解释*KRAS*基因突变在原发灶和异时性转移灶中不一致的原因。我们的数据表明，*KRAS*基因状态在转移过程中不总是保持稳定。一些NSCLC癌细胞在转移过程中丢失了*KRAS*突变基因型。尽管从原发肿瘤KRAS野生型到转移灶中突变型的转变，或是相反的情形都被观察到，但总体上原发灶*KRAS*基因突变频率明显高于转移灶。除部分NSCLC癌细胞在转移过程中丢失了*KRAS*突变基因型这一解释之外，也可能是KRAS野生型癌细胞早期更容易发生转移，而此时*KRAS*突变型克隆株癌细胞尚未发生转移。当然某些特定的基因型或许具有特定的器官转移亲嗜性，本研究纳入的转移灶部位不同，而不同转移部位癌细胞的异质性也有报道^[30, 31]^，尚需对此进行更进一步的研究。另一原因可能是受限于检测方法的敏感性和特异性，这涉及到异质性肿瘤内发生低频率的*EGFR*基因突变的检出率，目前尚缺乏*EGFR*和*KRAS*突变最佳的检测方法和标准。直接测序法检测突变DNA的阈值通常在20%至25%以上(以野生型DNA为背景) ^[19]^。通过测序确定的突变可以被视为病灶占优势的突变状态。结合突变富集PCR(mutant-enriched *PCR*, ME-PCR)和异源双链法分析法(heteroduplex analysis, HA)可以增加*EGFR*和*KRAS*突变检出的敏感性。Cortot等^[18]^研究显示，当应用ME-PCR这一更敏感的方法时，原发肿瘤和转移灶中*KRAS*突变状态的不一致被排除3例。尽管如此，其不一致率仍达到14%(3/21)。Park等^[21]^利用异源双链分析法分析原发灶和转移淋巴结的*EGFR*突变情况，检出率分别增加8例和15例，不一致率仍达15.8% (16/101)。显然*EGFR*和*KRAS*突变在原发灶和转移灶中的不一致性不能完全归于*EGFR*突变检测技术方法的局限性，即使*KRAS*突变只在一小部分细胞中检测出，也可能赋予他们抗酪氨酸激酶抑制剂特性。而同一患者中的不同区域和位置的肿瘤病灶对靶向治疗的反应也可能存在差异，需要进一步研究证实。

肿瘤异质性的研究^[32, 33]^显示，不同人类肿瘤的癌细胞群中存在广泛的细胞遗传学和表观遗传变异。激光捕获显微切割(laser capture microdissection, LCM)技术也被用来捕获混合组织标本中的纯瘤细胞以得到更精确的*EGFR*和*KRAS*突变分析结果。Reymond等^[34]^比较骨髓微转移和原发瘤*KRAS*突变时发现，播散的癌细胞不总是克隆于原发瘤灶。此外，循环血癌细胞和原发瘤在基因和蛋白表达上也存在相当大的异质性^[35, 36]^。本研究针对*EGFR*基因拷贝数和EGFR蛋白表达*meta*分析结果显示，原发灶和转移灶*EGFR*基因拷贝数和蛋白表达均存在较大差别，*EGFR*基因高拷贝数似乎更多见于转移灶中(RR=0.74, 95%CI: 0.53-1.02, *P*=0.06)。而原发灶中EGFR蛋白表达率多于转移灶(RR=1.13, 95%CI: 0.98-1.31, *P*=0.09)。原发灶和转移灶EGFR蛋白表达和基因拷贝数一致率分别为70.8%和72.9%。其中，Watzka等^[28]^通过尸检获得NSCLC原发灶和相应转移灶用以评价EGFR蛋白表达情况，尸检取材的优点是肿瘤组织足够分析，避免了活检组织取材不足所致假阴性。结果表明，即便如此原发肿瘤和转移瘤之间EGFR表达的不一致率仍达30%。临床研究^[37, 38]^显示，EGFR高拷贝数和*EGFR*突变相关，NSCLC中，*EGFR*突变较EGFR蛋白表达为一个更好的EGFR靶向治疗获益的预测指标^[39]^。研究还发现，在肺腺癌中*EGFR*基因的高拷贝数和基因突变激活并不总是伴随着EGFR蛋白阳性表达。*meta*分析数据显示，至少从间接的分布情况来看，原发灶和转移灶*EGFR*突变、*EGFR*基因扩增和蛋白表达三者之间似乎并无相关性。但受直接研究三者之间相关性的研究数量所限，本文并未做进一步分析评价。FLEX试验更新结果显示，EGFR和KRAS状态似乎均不是西妥昔单抗联合化疗临床获益的预测指标。尽管有研究^[9-11]^显示，*EGFR*基因扩增和蛋白表达水平也可以用来预测TKI治疗反应，但目前仍缺乏二者作为EGFR-TKI和EGFR单克隆抗体疗效预测指标的有力证据。

漏斗图直观显示部分指标略有不对称，考虑各项指标检测在原发灶和转移灶中状态不存在研究者和编辑喜好的“阳性”倾向性，结合失安全系数结果入选文献的发表偏倚不大。倒漏斗图不对称原因考虑为研究样本较少异质性明显所致，需要进一步扩大研究。

总之，针对目前有限的配对研究所做的系统评价结合当前研究，*EGFR*突变在NSCLC转移过程中似乎是以一种稳定的方式维持，而肺癌细胞的遗传异质性或许是导致*EGFR*基因状态部分不一致的原因。*KRAS*突变基因型早期即可出现，而在转移后有所减低，因而原发灶中*KRAS*基因突变更能反映出肺癌周身癌灶信息。联合检测原发灶中*EGFR*和*KRAS*基因突变可作为肺癌靶向治疗的疗效预测指标，单独对转移灶做KRAS状态分析可能会引入更多抵抗EGFR酪氨酸激酶抑制剂治疗的患者。鉴于*EGFR*基因高拷贝数在转移灶中所占的优势，或许在治疗选择时有必要对转移灶进行EGFR扩增分析以选择可能更适宜的NSCLC治疗患者，但具体临床选择仍需慎重。EGFR蛋白表达检测作为EGFR-TKI疗效预测指标目前仍缺乏足够的临床试验证据，其价值有待于进一步评估。若原发肿瘤显示EGFR高表达，针对转移灶进一步活检似乎没有必要，故不推荐对转移灶常规做EGFR蛋白表达的检测。

尽管如此，但鉴于总体研究样本量有限，仍然不能确定原发肿瘤和转移瘤中存在的分子异质性到底是肿瘤细胞转移扩散过程中遗传不稳定的结果还是肿瘤内异质性的结果。因此，仍需在扩大样本的前提下，采用更佳的检测方法来确定*EGFR*和*KRAS*基因状态在原发NSCLC和不同区域和部位转移灶的异质性，并观测TKI等靶向治疗药物的临床反应性，这对于EGFR靶向治疗策略选择具有重要意义。
